# Transcriptome Analysis in Cotton Boll Weevil (*Anthonomus grandis*) and RNA Interference in Insect Pests

**DOI:** 10.1371/journal.pone.0085079

**Published:** 2013-12-27

**Authors:** Alexandre Augusto Pereira Firmino, Fernando Campos de Assis Fonseca, Leonardo Lima Pepino de Macedo, Roberta Ramos Coelho, José Dijair Antonino de Souza Jr, Roberto Coiti Togawa, Orzenil Bonfim Silva-Junior, Georgios Joannis Pappas-Jr, Maria Cristina Mattar da Silva, Gilbert Engler, Maria Fatima Grossi-de-Sa

**Affiliations:** 1 Embrapa Recursos Genéticos e Biotecnologia, Brasília, Distrito Federal, Brazil; 2 Graduate Program in Cellular and Molecular Biology, Center of Biotechnology, Universidade Federal do Rio Grande do Sul, Porto Alegre, Rio Grande do Sul, Brazil; 3 Graduate Program in Biology Molecular, Universidade de Brasília, Brasília, Distrito Federal, Brazil; 4 Graduate Program in Genomic Sciences and Biotechnology, Universidade Católica de Brasília, Brasília, Distrito Federal, Brazil; 5 Plateau Microscopique, Institut National de la Recherche Agronomique, Sophia-Antipolis, France; U. Kentucky, United States of America

## Abstract

Cotton plants are subjected to the attack of several insect pests. In Brazil, the cotton boll weevil, *Anthonomus grandis*, is the most important cotton pest. The use of insecticidal proteins and gene silencing by interference RNA (RNAi) as techniques for insect control are promising strategies, which has been applied in the last few years. For this insect, there are not much available molecular information on databases. Using 454-pyrosequencing methodology, the transcriptome of all developmental stages of the insect pest, *A. grandis*, was analyzed. The *A. grandis* transcriptome analysis resulted in more than 500.000 reads and a data set of high quality 20,841 contigs. After sequence assembly and annotation, around 10,600 contigs had at least one BLAST hit against NCBI non-redundant protein database and 65.7% was similar to *Tribolium castaneum* sequences. A comparison of *A. grandis*, *Drosophila melanogaster* and *Bombyx mori* protein families’ data showed higher similarity to dipteran than to lepidopteran sequences. Several contigs of genes encoding proteins involved in RNAi mechanism were found. PAZ Domains sequences extracted from the transcriptome showed high similarity and conservation for the most important functional and structural motifs when compared to PAZ Domains from 5 species. Two SID-like contigs were phylogenetically analyzed and grouped with *T. castaneum* SID-like proteins. No RdRP gene was found. A contig matching chitin synthase 1 was mined from the transcriptome. dsRNA microinjection of a chitin synthase gene to *A. grandis* female adults resulted in normal oviposition of unviable eggs and malformed alive larvae that were unable to develop in artificial diet. This is the first study that characterizes the transcriptome of the coleopteran, *A. grandis*. A new and representative transcriptome database for this insect pest is now available. All data support the state of the art of RNAi mechanism in insects.

## Introduction

Insects comprise more than one million of the described species. Despite the great diversity of species and the importance of insects, mainly as disease vectors and agricultural pests, attainable molecular biology resources for insect study still need to be increased in order to understand their physiology and biochemistry and to find new targets for biotechnological tools. At least one-third of insect species are beetles, making coleopterans the most diverse order of living organisms. Nevertheless, few data are available for coleopteran molecular resources in the current available databases. The NCBI nucleotide database (http://www.ncbi.nlm.nih.gov/nuccore/), for example, has currently around 340,000 of total sequences for beetles. Only eight coleopteran species have more than 5,000 sequences deposited: *Pogonus chalceus* (65779), *Dendroctonus ponderosae* (41429), *Rhynchophorus ferrugineus* (27014), *Dendroctonus frontalis* (20987), *T. castaneum* (16808), *Ips* *typographus* (14810), *Agrilus planipennis* (12018), *A. grandis* (5705). Moreover, no more than 50 insect complete genomes are available for blast tool nucleotide search. From these, just one, *T. castaneum*, is coleopteran. Recently, a consortium called the i5k Initiative, also known as the 5,000 Insect Genome Project, was recently launched and aims to sequence the genomes of all insect species known to be important to worldwide agriculture, food safety, medicine, and energy production [1].

In the last years, the study of insect transcriptomes has been employed to evaluate gene expression profile for biotechnological use. After the introduction of Next Generation Sequencing techniques largely used for genome sequencing, transcriptome analysis became fast and efficient, and it is currently possible to detect and identify microRNAs, 3’ and 5’ untranslated regions and even complete mRNAs [2,3]. Particularly, the next generation sequencing strategy of pyrosequencing using 454 [4,5] has been used to study insect vectors [6–9] and to assess insect genes that code for hemolymph and midgut proteins [10–12], metabolic pathway enzymes [13–15] and metagenomics [16]. Moreover, some transcriptome studies were made in order to provide ESTs datasets of model and non-model insects [9,17–19] and have become an effective way of assessing gene expression levels. 

The cotton boll weevil, *A. grandis*, has been the most important cotton insect-pest in North America. Due to the Boll Weevil Eradication Program, sponsored by the USDA, an integrated pest management strategy has been successful in controlling boll weevil populations [20]. In South America, nevertheless, the insect populations are still causing great damage to the cotton crops, destroying cotton plant floral buds and bolls. Due to their high reproductive rate in tropical areas and to the endophytic behavior of earlier developmental stages, infestation levels increase fast and unless control measures are adopted, damages can lead up to total loss of production [21]. The ineffectiveness and harmful aspects in using chemical control to arrest the infestation leads to the search for more efficient control strategies, of which the most promising are in the biotechnological area.

The use of genetically modified (GM) crops to control insect pests is now widely used. Several proteins have been introduced in plants in order to control insects, mainly the *Bacillus thuringiensis* (*Bt*) toxins [22]. None is reported to control cotton boll weevil. The use of double-stranded RNA (dsRNA) to silence gene expression is currently a highly explored approach to generate insect-resistant genetically GM crops [23–25]. Moreover, RNAi is tool widely used in reverse genetics studies. Recent results showed the viability of the use of dsRNA-producing plants as an insect-pest control approach. Two groups reported GM plants that express dsRNA matching essential genes in the digestive tract of two important agricultural insect pests, the cotton bollworm *Helicoverpa armigera* (Lepidoptera) [26] and the western corn rootworm, *Diabrotica virgifera virgifera* (Coleoptera) [23]. In both cases, mortality was achieved after feeding on artificial diet containing dsRNA and GM plants expressing those dsRNAs had increased resistance towards the insects. These works support RNAi as a promising methodology for insect-pest control, making the search for candidate genes to be silenced an important step in control achievement.

RNA-mediated gene silencing as a mechanism was first described in plants as post-transcriptional gene silencing (PTGS) [27,28]. However, the discovery of the interference RNA mechanism (RNAi) in the free-living nematode *Caenorhabditis elegans* led to the understanding of the core characteristics of RNA-mediated gene silencing [29,30]. RNAi pathway is a natural cell mechanism in which mRNA-complementary dsRNA hybridizes specifically to mRNA leading to its degradation by enzyme complexes. The basic process seems to be conserved in the species studied so far. However, significant differences have been reported concerning the amplification and spread of systemic silencing signal and the silencing effect inheritance [25,31]. Opposite to *C. elegans*, the RNAi silencing effect in insects is restricted to the site of dsRNA delivery and endures shortly. So far, no gene was reported to be involved in a systemic mechanism for RNAi in insects, although studies have shown RNAi systemic effect in *T. castaneum* [32–34]. In this context, the sequencing of insect genomes and transcriptomes may provide more information about the genes involved in RNAi silencing pathway [35]. 

 In this work, analysis of more than 500,000 reads obtained by 454-pyrosequencing, assembled in 20,384 contigs is reported. Predicted proteins were compared to known insect genomes: *B. mori*, *T. castaneum* and *D. melanogaster*. Moreover, the analysis of contigs related to core interference RNA mechanism was performed by comparison to the RNAi insect genes. The sequences generated in this work will be a reliable source for candidate genes involved in essential physiological processes to be used in insect control using gene silencing via RNAi. In addition, dsRNA synthesized using *A. grandis* chitin synthase 1 gene as a template was delivered to cotton boll weevil female adults and managed to trigger chitin synthase 1 silencing. 

## Materials and Methods

### Insects

Eggs, larvae and adult cotton boll weevils were reared in artificial diet according to Monnerat et al [36] at the Laboratório de Bioecologia e Semioquímicos de Insetos of Embrapa Recursos Genéticos e Biotecnologia in Brasília, Brazil. The insects were kept at 26 ± 2 °C, 60 ± 10% relative humidity and 12 h:12 h light:dark. Larvae instars were determined by measuring head capsule width, as described for lepidopterans [37]. Adult sex determination was performed according to Sappington and Spurgeon [38].

### RNA purification, cDNA library construction/normalization and pyrosequencing

Total RNA was extracted separately from each insect stage, eggs, larvae (3 instars), pupae and male and female adults using Trizol Reagent (Invitrogen Life Technologies), according to the manufacturer. RNA was treated with RNAse-free DNAse I (Ambion, Invitrogen Life Sciences) at 37 °C for 30 minutes, according to the manufacturer. A pool of 30µg of all insect stages total RNA was sent to synthesize a cDNA library at Eurofins MWG Operon, in Huntsville, AL, USA (http://www.eurofinsdna.com/). 

The RNA quality was assessed in a Agilent 2100 Bioanalyzer before cDNA library construction. Full-length, enriched, cDNAs were generated using the SMART PCR cDNA synthesis kit (BD Clontech) following the manufacturer’s protocol. In order to prevent over-representation of the most common transcripts, the resulting double-stranded cDNAs were normalized using the Kamchatka crab duplex-specific nuclease method (Trimmer cDNA normalization kit, Evrogen) [39]. Normalized cDNA was submitted to half-plate run 454 pyrosequencing, GS FLX Titanium technology, according to protocols provided by manufacturer (Roche 454 Life Sciences). 

### Pre-processing

Pyrosequenced reads were pre-processed using *est2ssembly* 1.03 pipeline [40]. Contaminant sequences (prokaryotic, viral, mitochondrial sequences) were removed after BLAST analysis. Transcriptome data was deposited in NIH Short Read Archive, with accession number SRA059959.

### Assembly, Annotation and Gene Ontology (GO)

Contigs were assembled using MIRA v3.3.0.1 [41]. Homology searches (BLASTX and BLASTN) of unique sequences and functional annotation by GO terms (www.geneontology.org), InterPro entries (InterProScan; http://www.ebi.ac.uk/Tools/pfa/iprscan/), enzyme classification codes (EC) and metabolic pathways (KEGG, Kyoto Encyclopedia of Genes and Genomes; http://www.genome.jp/kegg/) were determined using Blast2GO software suite v2.4.3 (www.blast2go.org). Sequences were submitted to NCBI protein nr databank via BLASTx, with e-value threshold of 10^-5^. False Discovery Rate (FDR) was used at 0.05% probability level. GO terms were improved with ANNEX tool [42], followed by GOSlim tool available at Blast2GO (goslim_generic.obo) [43]. Combined graphs were constructed at levels 2, 3 and 5 for Biological Process, Cellular Component and Molecular Function categories, respectively. Enzymatic classification codes and KEGG metabolic pathways were generated of direct mapping of GO terms, with their respective ECs. InterPro searches were performed remotely from Blast2GO on InterProEBI server. 

A comparison of cotton boll weevil transcriptome to *Tribolium castaneum* genome annotation was carried out using WEGO tool (Web Gene Ontology Annotation Plotting - http://wego.genomics.org.cn) [44].Venn Diagram was constructed using a free tool found in the Bioinformatics and Evolutionary Genomics Laboratory website, hosted by the University of Gent Plant System Biology Department (http://bioinformatics.psb.ugent.be/webtools/Venn/).

### Sequence alignment, SID phylogenetic analysis and in silico analysis of PAZ Domain contigs

Textual and sequence similarity searches were performed in the transcriptome database for genes involved in RNAi mechanism, based on available NCBI Protein Database sequences (http://www.ncbi.nlm.nih.gov/protein). 

The amino acid sequences of PAZ domains and SID proteins were obtained by *in silico* translation using TrEMBL (http://www.expasy.ch/tools/dna.html). 

Two largest PAZ Domain-containing contigs, here called *A_grandis_454_c1018* and *A_grandis_454_c4142* were selected for alignment with PAZ domains of argonautes and dicer-like proteins of other organisms including insects (Figure S1A). All sequences used for alignment contained full PAZ Domains, including *A. grandis* contigs and were submitted to ClustalW2 Multiple Sequence Alignment (http://www.ebi.ac.uk/Tools/msa/clustalw2/) [45] and edited with Jalview tool (http://www.jalview.org/) [46].

For SID-like protein analysis, a complete gene sequence of *A. grandis* A_grandis_454_c2889 was translated. Sequence alignment was carried out using complete protein sequences (Figure S1B). Full SID proteins were aligned using Clustal W [47]. Phylogenetic and molecular evolutionary analyses were conducted using MEGA version 5 [48]. The results of alignments were used for constructing neighbor-joining trees with bootstrap analysis of 10,000 replicates and evolutionary divergence calculated by p-distance method.

### Chitin Synthase dsRNA bioassay

 Chitin Synthase 1 contig (AntgCHS1) was searched in *A. grandis* transcriptome using tBLASTx. A specific fragment of 253 bp was chosen using BLOCK-iT™ RNAi Designer (http://rnaidesigner.invitrogen.com/rnaiexpress/). The fragment was amplified with primers 5’ATCACAGGAGCAGCGTTGC and 3’ACACCAACTTATCCAATATC, both containing T7 promoter minimum sequence at 5’ end. PCR product was cloned to pGEMT-easy vector (Promega) and sequenced to verify correct amplification. dsRNA was synthesized with 0,5 µg of PCR product as template, using MEGAscript® T7 High Yield Transcription kit (Invitrogen). AntgCHS1 dsRNA was dissolved in DEPC-treated water and quantified by spectrophotometry. 

Female adults aged 48 hours were microinjected with 1 µL of 200 ng AntgCHS1 dsRNA before copulation, using a 10 µL Gastight Luer connection (LT) syringe (1701LT model), with a 51 mm, gauge 26S, point style 4, 12° bevel needle. Each experimental unit consisted of 16 female adults, which, after microinjection were kept in a sieved box with 8 non-injected males, in order to allow the collection of laid eggs. Males were previously marked with ink. The sieved box with insects contained artificial diet [36] and was maintained at 26 ± 2 °C, 60 ± 10% relative humidity and 12/12-hour day/night photoperiod. Control was performed by microinjection of *E. coli gus* gene dsRNA, produced as previously described. Analyzed phenotypic parameters were oviposition, egg viability, and adult mortality. Eggs laid by microinjected females were mechanically pierced and kept in artificial diet for 7 days. Neonate larvae development and phenotypes were assessed. Data of three bioassays were applied to variance analysis and Tukey’s multiple comparisons test at 5% level of significance.

## Results and Discussion

### Sequencing, Assembly and Annotation

**Figure 1:**
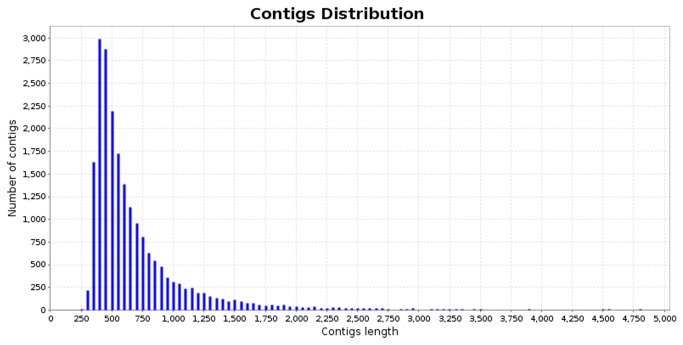
Contigs length distribution showing the major number ranging from 350 to 1000 pb. The average contig length was 676pb.

A cDNA library was constructed after pooling RNA extracted from boll weevil eggs, three larval instars, pupae, male and female adults. cDNA-normalized library 454 runs generated a total of 576,297 ESTs (Table 1). The minimal quality standards used in pre-processing provided 310,182 reads, with average read length of 379 bp. These data were deposited in NIH Short Read Archive, with accession number SRA059959. After assembly, 20,384 contigs with average length of 676bp were obtained, with an average depth of coverage of 9.58 sequences per nucleotide position. Of these, most contigs have length ranging from 300 to 750 bp (Figure 1).

**Table 1:** Summary of *Anthonomus grandis* transcriptome sequencing assembly and annotation.

**Number of reads before pre-processing**	576,297
**Number of bases before pre-processing**	179,676,724
**Average read length before pre-processing (bp)**	379
**Number of reads after pre-processing**	310,182
**Number of bases after pre-processing**	119,094,383
**Average read length after pre-processing (bp)**	383
**Number of contigs**	20,384
**Number of bases in contigs**	13,780,583
**Average contig length (bp)**	676
**Min. contig length (bp)**	201
**Max. contig length (bp)**	4,847
**Average read coverage per contig**	9,58
**% contigs with at least 1 IPR**	70
**Contigs with at least 1 blast hit against nr**	10,621

### Similarity searches and gene ontology analysis

After read assembly, contigs were submitted to BLASTx similarity search against NCBI non-redundant protein database (nr) to assess their putative function. Around 10,600 contigs showed at least one hit against nr (Table 1). Of these, 84.9% showed significant blast matches at a cutoff e-value ≤ 10^-3^ (Figure S2). Contigs with e-value = 0 were represented at the end of the figure, and correspond to 2.5% of the total number of contigs.

**Figure 2:**
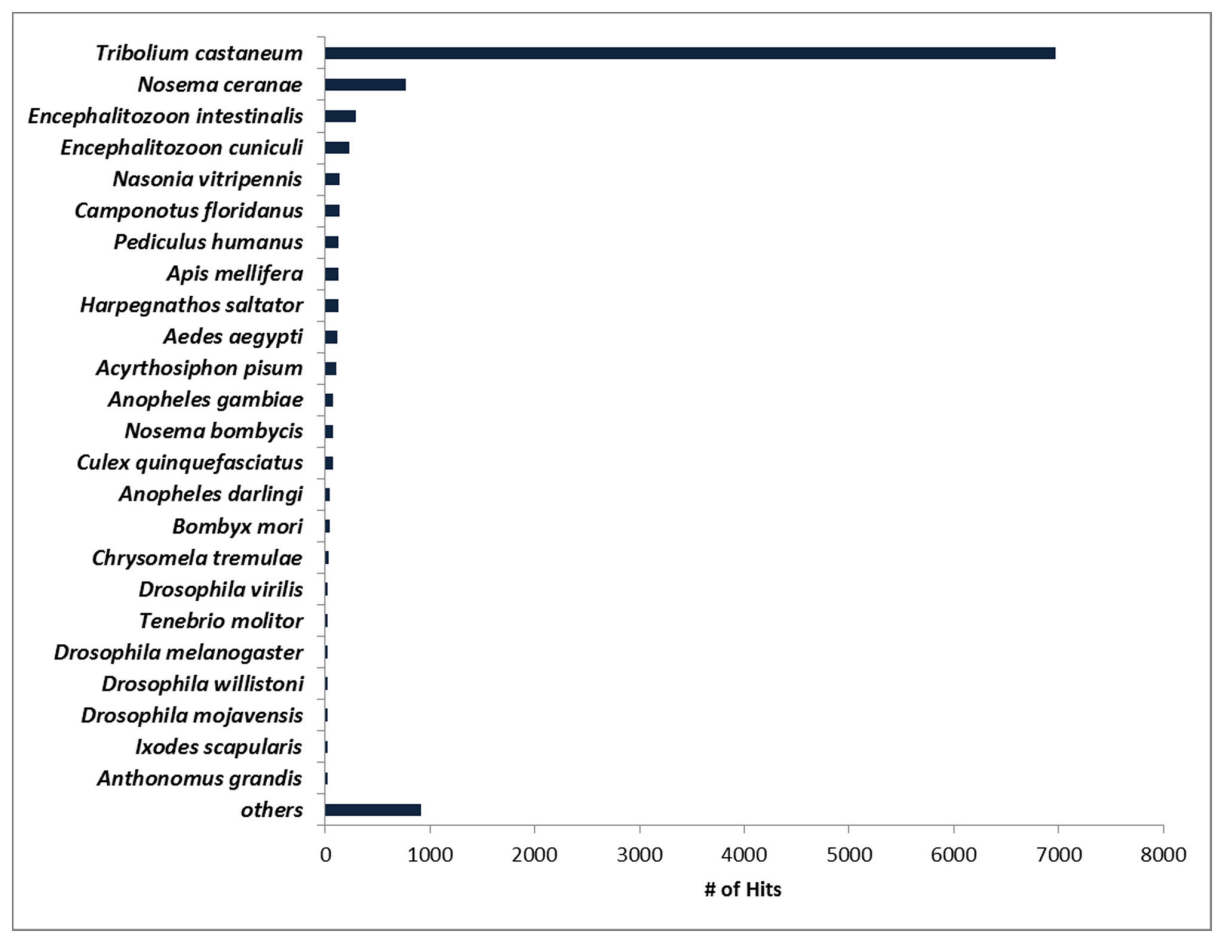
Species distribution of top BLASTx matches of *A. grandis* contigs. A great number of contigs matched insect genes, mainly another coleopteran, *T. castaneum*. E-value cutoff is 1x10^-3^.

Figure 2 shows the top-hit species after BLASTx similarity search. As expected, 65.7% of the contigs were similar to *T. castaneum* sequences. *T. castaneum* (red flour beetle) is the most important coleopteran of *Tenebrionidae* family because it attacks stored grain products and is responsible for great loss and damage. Until now, it is the only coleopteran with a fully sequenced genome available [49], which explains the far greater number of contigs of *A. grandis* with similarity to *T. castaneum* sequences. The three top matching species after *Tribolium* are fungi. Insect transcriptome pyrosequencing reports show a number of contigs of the Phylum Microsporidia. *Nosema* is a genus of microsporidian known to parasite a great number of arthropods. Insect orders parasited include Orthoptera, Lepidoptera, Diptera, Hymenoptera and Coleoptera. It is a very common contamination in boll weevil colony rearing and is found in the insect midgut [50]. The most studied species so far, with a whole sequenced genome, is *Nosema ceranae*, which cause a disease in honey bees and thus great loss to apiculture. The microsporidian genus *Encephalitozoon* is also described associated in symbiosis to insects [51,52]. Very important in human health, seven genome-sequencing projects of three *Encephalitozoon* species are deposited in the NCBI Genome Bank. Our data suggest that our insect colony was possibly infected by those microsporidians and some of their ESTs sequenced and well annotated due to the great number of available sequences on databases. 

The most part of ESTs was similar to insect sequences. Besides the coleopteran *T. castaneum*, the other insect species with full genome sequences, although phylogenetically distant, are distributed into the orders Hymenoptera (*Nasonia vitripennis*, *Camponotus floridanus*, *Apis mellifera*, *Harpegnathos saltator*), Phthiraptera (*Pediculus humanus*), Diptera (*Aedes aegypti, Anopheles gambiae*, *Culex quinquefasciatus*, *Anopheles darlingi*, *Drosophila virilis*, *Drosophila willistoni*, *D. melanogaster, Drosophila mojavensis*), Hemiptera (*Acyrthosiphon pisum*), and Lepidoptera (*B. mori*). The coleopterans *Tenebrio molitor* and *Chrysomela tremulae* also were among the top-hit species, but with a low number of matched contigs, probably because they do not have their genomes sequenced yet. This may also explain why *A. grandis* has a low number of matched sequences.

The *A. grandis* transcriptome was GO-annotated based on matches to Interpro proteins. In order to group the proteins with associated GO terms, the top level terms for each GO category "Molecular function", "Biological Process" and "Cellular component" were recorded at the different match levels. The dominant terms for Molecular function are clearly transporter activity and binding, while the dominant term for Biological process is pigmentation. Within Cellular component the dominant terms are evenly divided between organelle, cell part and organelle part (Figure S3A, B and C).

A more detailed classification of the contigs function can be obtained from the top 35 InterPro entries (Table 2). The most abundant entry is NAD(P)-binding domain (IPR016040). Chaperones, nucleic acid binding and oxidative stress-related domains constitute the most part of InterPro entries, in accordance to the grouped GO top terms (Figure S3A, B and C).

**Table 2:** Main protein families found in cotton boll weevil transcriptome.

**InterPro Entry Accession**	**# of Contigs**	**InterPro Entry Name**
IPR016040	154	NAD(P)-binding domain
IPR011009	145	Protein kinase-like domain
IPR016196	116	Major facilitator superfamily domain, general substrate transporter
IPR011046	110	WD40 repeat-like-containing domain
IPR015943	101	WD40/YVTN repeat-like-containing domain
IPR015880	94	Zinc finger, C2H2-like
IPR012677	88	Nucleotide-binding, alpha-beta plait
IPR016024	84	Armadillo-type fold
IPR000504	83	RNA recognition motif domain
IPR001680	79	WD40 repeat
IPR012336	77	Thioredoxin-like fold
IPR007087	73	Zinc finger, C2H2
IPR017853	73	Glycoside hydrolase, superfamily
IPR002198	67	Short-chain dehydrogenase/reductase SDR
IPR013781	67	Glycoside hydrolase, subgroup, catalytic domain
IPR009003	66	Peptidase cysteine/serine, trypsin-like
IPR001254	65	Peptidase S1/S6, chymotrypsin/Hap
IPR011992	59	EF-hand-like domain
IPR001650	55	Helicase, C-terminal
IPR000618	54	Insect cuticle protein
IPR001128	54	Cytochrome P450
IPR001611	49	Leucine-rich repeat
IPR002290	45	Serine/threonine- / dual-specificity protein kinase, catalytic domain
IPR002018	44	Carboxylesterase, type B
IPR009057	44	Homeodomain-like
IPR011989	44	Armadillo-like helical
IPR011990	44	Tetratricopeptide-like helical
IPR016027	44	Nucleic acid-binding, OB-fold-like
IPR015424	42	Pyridoxal phosphate-dependent transferase, major domain
IPR003959	41	ATPase, AAA-type, core
IPR012340	41	Nucleic acid-binding, OB-fold
IPR002557	40	Chitin binding domain
IPR009072	40	Histone-fold
IPR011701	40	Major facilitator superfamily
IPR001353	39	Proteasome, subunit alpha/beta

**Figure 3:**
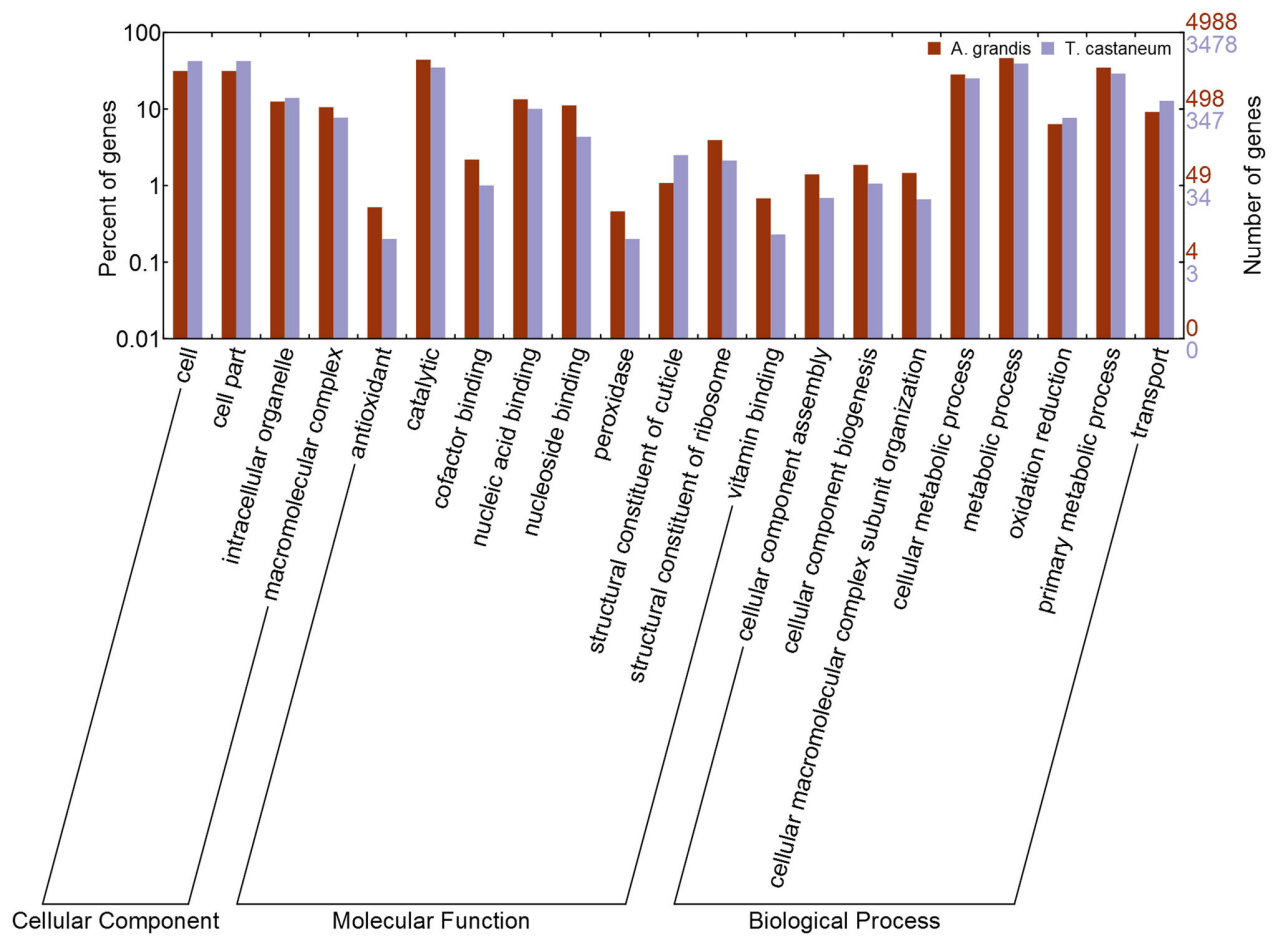
Comparison of the distribution of GO terms. The X-axis shows subgroups of cellular component, molecular functions and biological process from GO. Distribution of GO terms of gene families of *T. castaneum* and *A. grandis* are compared. The Y-axis shows the percentage (left) and the number of genes (right) of the matched Pfam entries.

We used WEGO [44] for visualizing and comparing our GO annotation to the *T. castaneum* genome annotation data (Figure 3). Similar number of genes was annotated for the same GO terms in both insects for a determined GO category and no significant differences were shown, which indicates that *de novo* annotation for *A. grandis* is comparable to the *T. castaneum* genome annotation. Hence, we consider that we accomplished the objective of generating a database describing a significant and representative portion of the *A. grandis* transcriptome.

**Figure 4:**
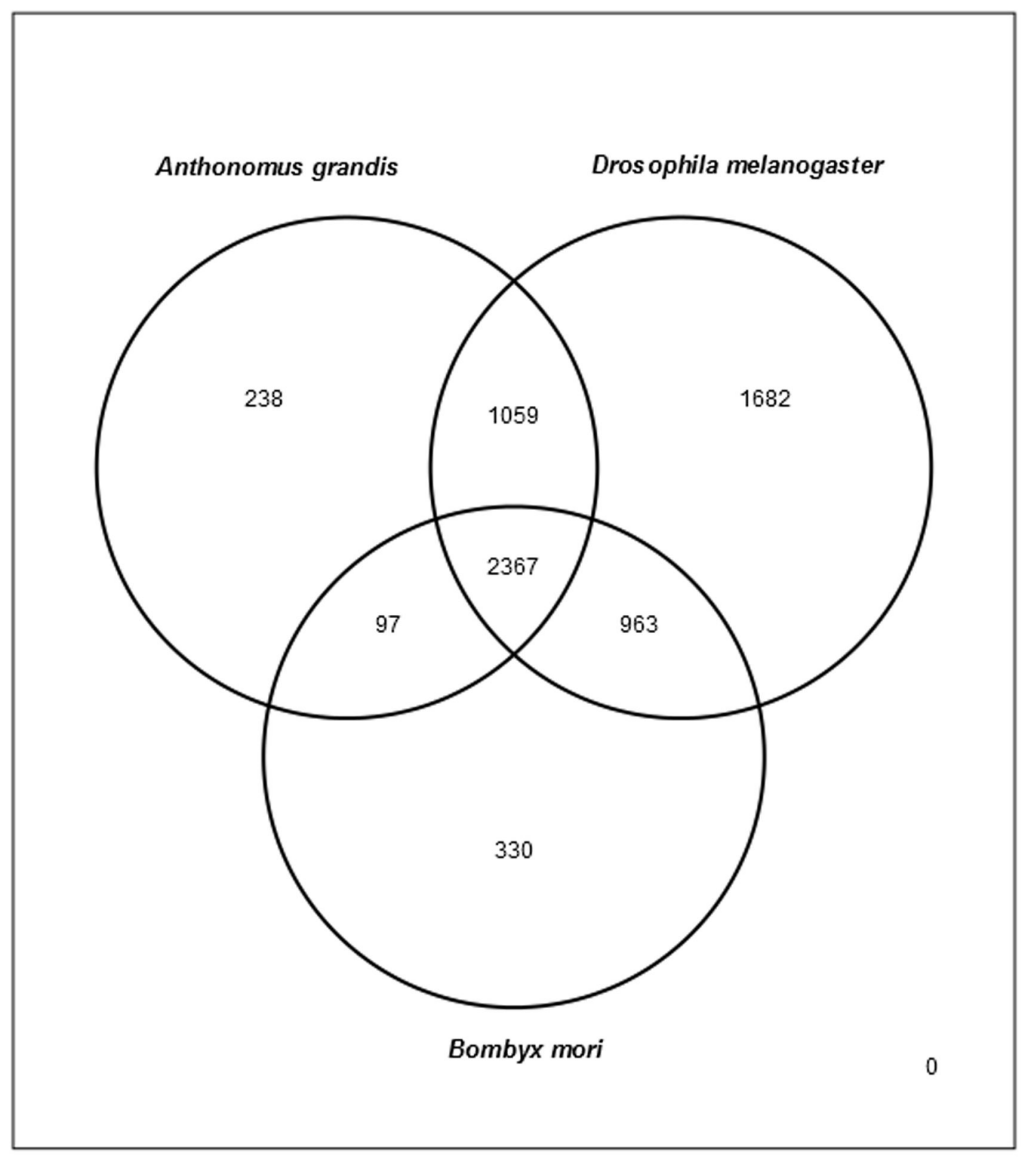
Venn diagram of the number of contigs from *A. grandis* which show IPR matches to *D. melanogaster* and/or *B. mori*. Numbers are unique Butterflybase and Flybase IPR results. The number of similar protein families between *A.grandis* and *D. melanogaster* is higher than *A.grandis* and *B. mori*.

 We performed a comparison of the *A. grandis* 454 Pfam entries to *D. melanogaster* and *B. mori* Pfam transcript sects from Flybase [53] and Silkbase [54] (with tBLASTx, e < 10^-3^) in order to establish a simplified genetic overlap between these species. The low number of *A. grandis* sequences, which do not match either *D. melanogaster* or *B. mori* (Figure 4) is probably due to the sum of new unique genes, poorly conserved genes, and erroneously sequenced reads. We noticed that the protein family similarity is higher to *Drosophila* (Diptera) than to *Bombyx* (Lepidoptera). This is significant because the number of sequence data in plant-insect pest interaction is greater for Lepidoptera than for Diptera, which normally lead to a probably erroneous biased search for ortholog sequences for coleopterans in lepidopteran databases. 

### Proteins involved in RNA interference mechanism

**Figure 5:**
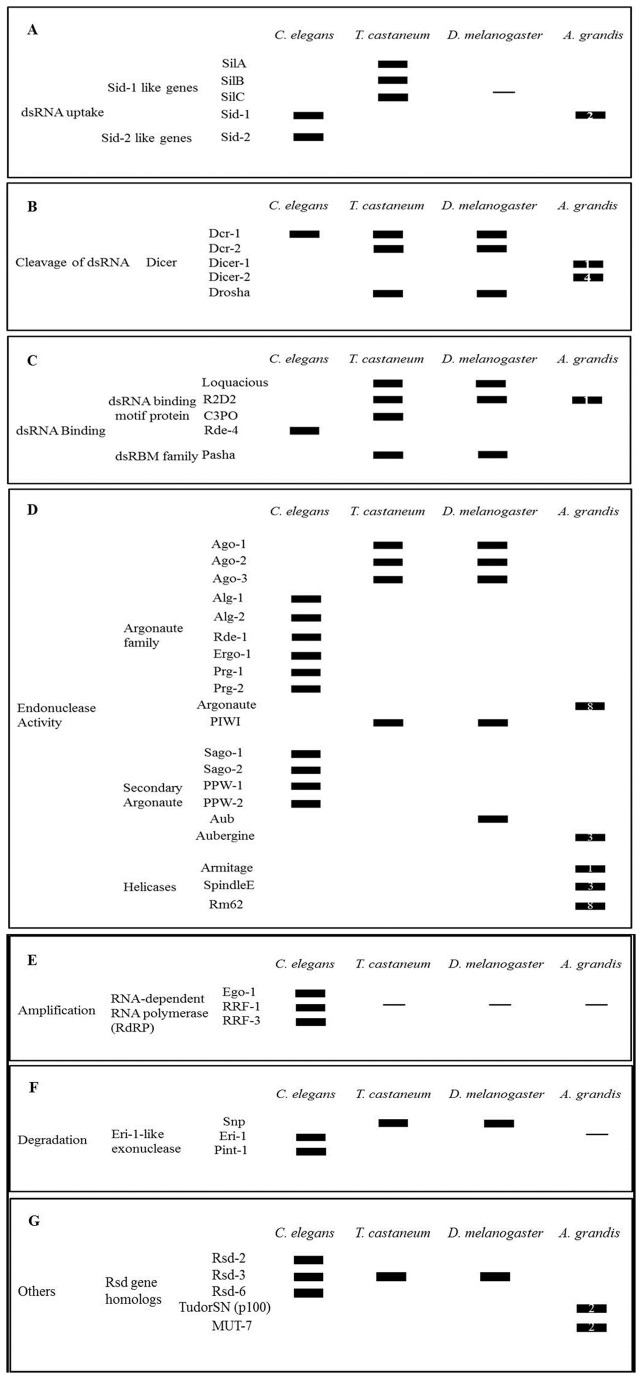
Genes involved in RNAi mechanism found in *A. grandis* transcriptome. The comparison with genes of *C. elegans*, *T. castaneum*, and *D. melanogaster* suggested that RNAi mechanism is well conserved in insects (A, B, C, D), including lack of amplification (E). No gene involved in dsRNA degradation was found (F). The number of contigs found in *A. grandis* transcriptome for each gene class is shown.

The mechanisms of RNAi seem to be conserved among species, despite the previously described differences regarding signal amplification, systemic effect and inheritance [32]. In insects, except dipterans, dsRNA uptake is carried out by SID-1. Once inside the cell, dsRNA is cleaved in small RNAs (siRNAs) by Dicers. siRNAs are recognized by the RNA-induced silencing complex (RISC), which contain argonaute proteins. The siRNAs hybridize with specific mRNAs and the duplex siRNA-target mRNA is then degraded. We have found several contigs of genes coding for proteins involved in RNAi mechanisms (Figure 5). Most proteins sequenced belonged to Argonaute, Dicer and Helicase families, involved in dsRNA cleavage and endonuclease activity. The number of contigs found for each gene class is indicated. 

Based on the contigs found, RNAi mechanism in *A. grandis* seems to be similar to other insects in the steps of the process like dsRNA cleavage, dsRNA binding and Argonaute activity (Figure 5B, C, D), but differs of dipterans in dsRNA uptake (Figure 5A). No gene involved in dsRNA degradation was found (Figure 5F). The contigs found best matched insect genes, mainly from dipteran and coleopteran species (Table S1). 

Two *sid-1* contigs (A_grandis_454_c14864, A_grandis_454_rep_c2889, 709bp and 1918bp, respectively), gene that codes for the membrane protein responsible for dsRNA uptaking and spreading through the tissues, were found. The top species BLASTx hit for these two contigs was *T. castaneum*, which has three *sid-1* paralogs in its genome. Both contigs have above 60% identity and e-value < 5x10^-31^. Those contig sequences do not overlap, and probably are paralog genes. Their best BLASTx hits are *T. castaneum sid-1A* and *sid-1C*, respectively. 

**Figure 6:**
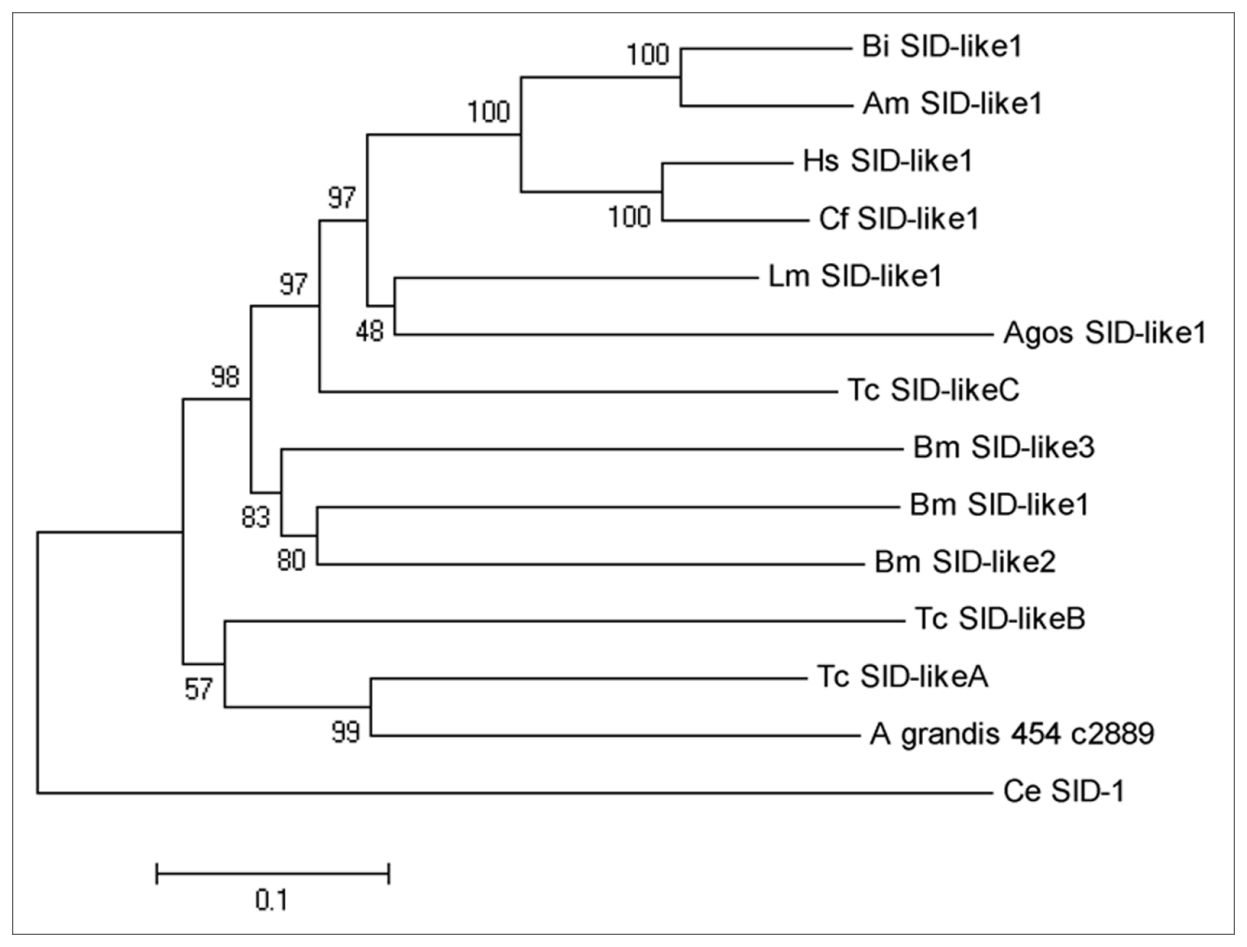
Distance neighbor-joining tree showing the phylogeny of a SID-like contig of *A. grandis* (A_grandis_454_c2889) and SID-like proteins of the insects *T. castaneum*, *B. impatiens*, *A. mellifera*, *L. migratoria*, *B. mori*, *A. gossypii*, *H. saltator*, *Camponotus floridanus*. The percentage of percentage of bootstrap confidence values is shown at the nodes.

We used the predicted protein from contig A_grandis_454_c2889 for phylogenetic analysis because it contains the complete ORF for *sid-1*. A distance/neighbor-joining dendrogram for the SID proteins grouped the A_grandis_454_c2889 contig with SID-like A and SID-like B from *T. castaneum* (Figure 6). SID-like C from *T. castaneum* is closer to hemipteran *A. gossypii* and grouped in the branch that have homopteran and mainly hymenopteran insects. Probably, the contig A_grandis_454_c14864 that has as BLASTx best hit *sid-1C* of *T. castaneum*, could group in the same branch, although we need full gene sequence to confirm it. An evaluation of available genomes shows that the number of *sid-1* gene copies varies among insects. Dipterans have no *sid-1* genes, and hemipterans, hymenopterans, orthopterans and phthirapterans have just one *sid-1* [35]. Among lepidopterans this number are even more variable: while *B.mori* has three, *Spodoptera exigua* has only one [25,35]. As previously described for other insects, no *sid-2* ortholog gene, which is present in nematodes, was found for *A. grandis*. 

No ortholog gene for RNA-dependent RNA polymerase (RdRP), the enzyme that amplifies RNAi signal in nematodes, was found (Figure 5E). Recent studies performed and patented by our group showed that delivery of dsRNA by microinjection was capable of trigger silencing of laccase2 [55] and chitin synthase 2 [56] genes in *A. grandis*. Since the morphological effects were observed far from the local of microinjection, this corroborates the already proposed hypothesis that when RNAi signal amplification occurs in insects, mainly in coleopterans, it may be mediated by other mechanism [57].

**Figure 7:**
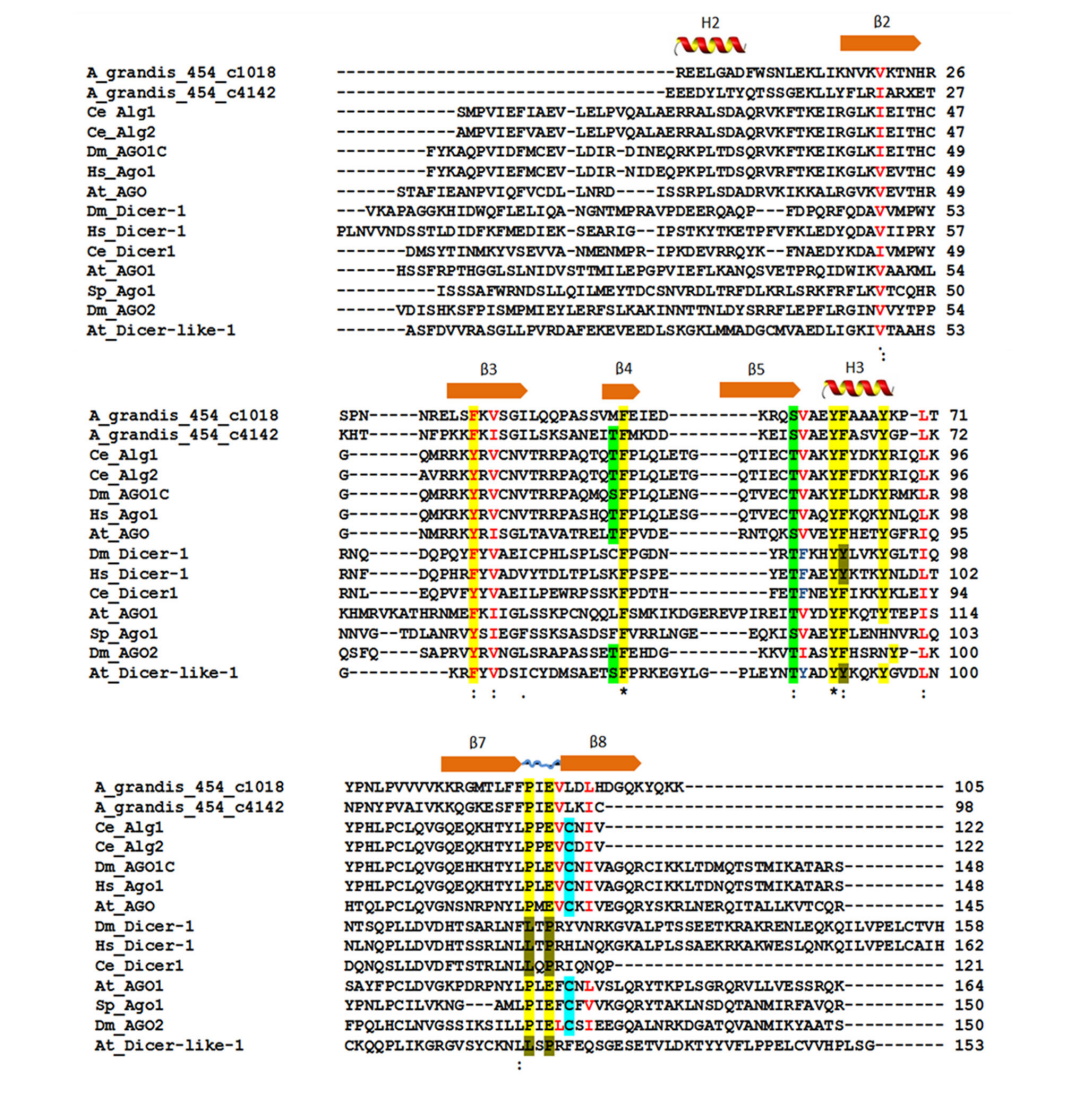
Comparison of dicer and argonaute PAZ domains. Two cotton boll weevil contigs were aligned to five species sequences: *D. melanogaster* (Dm_Dicer-1, Dm_AGO1C, Dm_AGO2)*, C. elegans* (Ce_Dicer1, Ce_Alg1, Ce_Alg2)*, Homo sapiens* (Hs_Dicer-1, Hs_Ago1)*, A. thaliana* (At_Dicer-like-1, At_AGO, At_AGO1) and *Schizosaccharomyces pombe* (Sp_AGO1). The sequence IDs are the same found in the NCBI Protein Database. Secondary structures within the domain are indicated as α-helices and β structures. The highlighted residues are responsible for the stabilization of the dsRNA-binding region. In yellow, a subdomain of aromatic residues. Along with a cysteine residue (blue), preceded by a proline and a glutamate (yellow), some invariant residues (red) create a hydrophobic subdomain that interacts with RNA. Residues that differ in dicer and argonaute PAZ domains are shown in brown.

 In order to evaluate a conserved domain in a protein involved in RNAi mechanism, we performed an alignment of the PAZ domains of two contigs from the boll weevil transcriptome (A_grandis_454_c1018 and A_grandis_454_c4142) with dicers and argonautes of 5 different species: (Figure 7): *D. melanogaster* (Dm_Dicer-1, Dm_AGO1C, Dm_AGO2)*, C. elegans* (Ce_Dicer1, Ce_Alg1, Ce_Alg2)*, Homo sapiens* (Hs_Dicer-1, Hs_Ago1)*, Arabidopsis thaliana* (At_Dicer-like-1, At_AGO, At_AGO1) e *Schizosaccharomyces pombe* (Sp_AGO1). PAZ is a double-stranded-RNA-binding domain present in all argonautes and dicers [31,58]. Conserved residues in dicers and argonautes are also present in *A. grandis* contigs, which can validate in transcriptome assembly. These residues are normally located on the domain surface and at only one side of the RNA-binding proteins [59]. In figure 7, the highlighted residues are responsible for the stabilization of the dsRNA-binding region, forming seven β structures and a α-helix. A subdomain featuring aromatic residues (in yellow) keep the domain folding which is similar to an OB-fold (OB – Oligonucleotide/oligosaccharide Binding fold), known to bind single-stranded DNA unspecifically [60,61]. Along with a cysteine residue (blue), preceded by a proline and a glutamate (yellow), some invariant residues (red) create a hydrophobic subdomain that interacts with RNA. Differential residues between PAZ domains of dicers and argonautes suggest that the cotton boll weevil contigs belong to the latter family (in brown), although experimental approaches are necessary to confirm it. 

### Effect of parental AntgCHS1 dsRNA on eggs and neonate larvae

**Figure 8:**
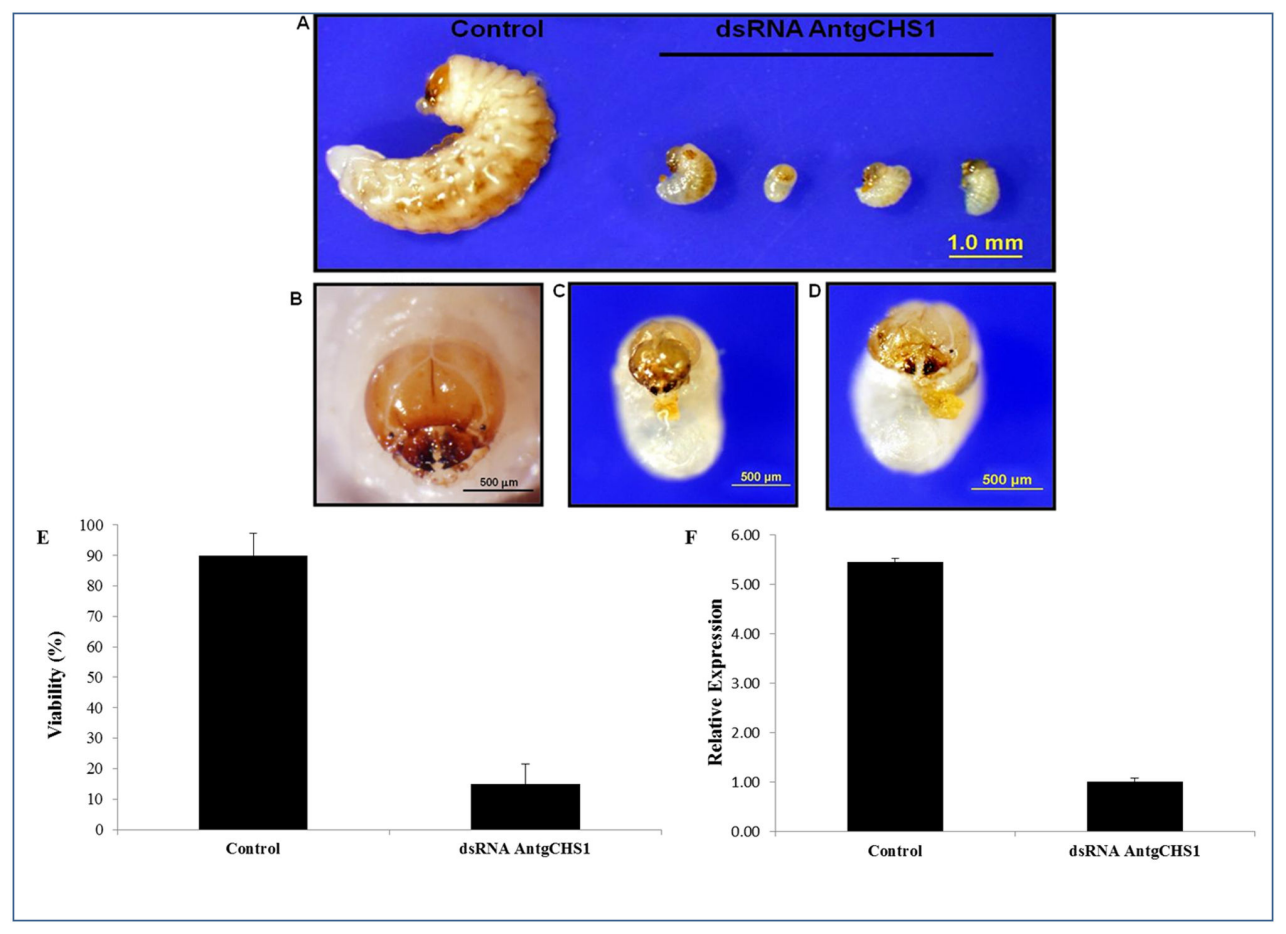
Effect of AntgCHS1 on *A. grandis* on oviposition. Larvae that emerged from eggs laid by females previously microinjected with 200 ng of either GUS (control) or AntgCHS1 dsRNA (A). After egg hatching, larvae were fed in artificial diet for 7 days. Details of head capsule show malformations in AntgCHS1 dsRNA-treated larvae (C and D) when compared to control (B). The viability was reduced (E) and as well as the number of transcripts of AntgCHS1 (F) in eggs laid by females previously microinjected with AntgCHS1 dsRNA.

 Chitin synthase (EC 2.4.1.16) is the final enzyme of the chitin synthesis pathway which polymerizes chitin by promoting covalent bonds between activated UDP-N-acetylglucosamine monomers [62]. Gene silencing reports have showed the importance of chitin biosynthesis for insect cuticle formation [63,64]. A chitin synthase contig was found in *A. grandis* transcriptome and here called AntgCHS1, corresponding to chitin synthase 1, enzyme described to trigger chitin polymerization in insect cuticle [62,65]. In order to evaluate the effect of RNAi gene silencing on *A. grandis*, GUS dsRNA and AntgCHS1 dsRNA were synthesised and delivered to female adults by microinjection before copulation. No effect was phenotipically observed in the microinjected females. AntgCHS1 dsRNA microinjection caused no effect in female survival. After copulation, the number of laid eggs was not different between treatments, but viability, measured as the average number of eggs which hatched and generated well-formed larvae, was reduced 84% for eggs laid by AntgCHS1 dsRNA (Figure 8E). Interestingly, embryo formation and normal movement inside the same eggs were observed, suggesting that larvae were formed but could not eclose. So mechanical perforation of egg shell was performed and larvae transferred to artificial diet and observed for seven days. Larvae from GUS dsRNA-microinjected females developed normally, while larvae from AntgCHS1 dsRNA-treated females failed to develop and died (Figure 8A). This can be explained by the observed head capsule and mandibule malformation which must have hampered diet feeding as well the previously described difficulty of tearing the egg shell and eclosing (Figure 8B, C, D). Previous studies have already reported the incapacity of egg shell rupture by larvae in which chitin synthesis was compromised [66]. Mutations in *D. melanogaster* chs *1* gene, formerly called *kkv*, caused the embryos to develop normally, but to fail in eclode from the eggs. When the vitelline membrane in these mutant eggs was punctured by mechanic pressure, embryos were alive and more stretched than wild-type embryos. This phenotype, called *blimp*, was explained by the failure of epidermal cells in synthesize the cuticle correctly. Loss of functionality in chitin synthases, either by mutation or by the use of synthetic inhibitors, like benzoylphenyl urea (BPU) can produce the same results [67–69].

In addition, eggs laid by microinjected females after copulation were used to evaluate the number of AntgCHS1 gene transcripts. The microinjection of 200 ng of AntgCHS1 dsRNA in adult females resulted in a 5,5-fold reduction of AntgCHS1 gene transcripts in eggs when compared to control, indicating that RNAi effect was transferred to the next generation (Figure 8F). These results confirm that synthesis of chitin in insect epidermis is affected in *A*. *grandis* after AntgCHS1 dsRNA delivery. Parental RNAi effect transferred to offspring was also reported for *T. castaneum* genes [32,34]. As discussed before, these facts also support the theory of at least one unknown mechanism of RNAi signal amplification, which is different from nematodes and plants, since insects do not have RdRP genes in their genomes. 

## Conclusions

Here it is described the analysis of a new database of cotton boll weevil (*A. grandis*) nucleotide sequences obtained by pyrosequencing of the insect transcriptome. It is the largest number of sequences provided for this insect pest so far. These results provide a significant molecular biology dataset, which can be used, as an example, for molecular prospection in order to validate genes to be used in insect control. The silencing of a chitin synthase gene in larvae emerged from eggs laid by dsRNA-microinjected females proved that not only RNAi machinery is able to trigger RNAi silencing in *A. grandis*, but also to transfer its effect to the next generation. Since the main goal here was to generate and analyze data *in silico*, other experiments of gene expression quantitation, silencing via RNAi and gene sequencing in specific insect stages or submitted to certain conditions must be carried out. These experiments will allow the characterization of processes, either to understand cotton boll weevil biology or to assess gene candidates for development of insect control biotechnological tools. 

## Supporting Information

Figure S1 **Orthologous genes used in PAZ Domain alignment (**A**) and SID-1 phylogenetic Analysis (**B**).** Two largest cotton boll weevil PAZ Domain-containing contigs were selected for alignment with PAZ domains of argonautes and dicer-like proteins of other organisms including insects. For SID-like protein phylogenetic analysis, a cotton boll weevil complete gene sequence was translated and aligned to complete protein sequences.
(TIF)

Figure S2 **E-value for the top BLASTx hits.** Sequences with e-value equal to 0 are represented in a peak at right. 84.9% of the contigs showed significant blast matches at a cutoff e-value ≤ 10^-3^.
(TIF)

Figure S3 **Gene ontology (GO) categories for *A. grandis* transcriptome.** The terms were classified on level 2, 3 and 5 in the (A) *Biological* *Process*, (B) *Cellular* *Component* and (C) *Molecular* *Function*, respectively. The dominant terms for Molecular function are transporter activity and binding, while the dominant term for Biological process is pigmentation. Within Cellular component the dominant terms are evenly divided between organelle, cell part and organelle part. The percentage of contigs in each GO term is shown.
(TIF)

Table S1 ***A. grandis* contigs found in the transcriptome corresponding to RNAi insect genes.** RNAi mechanism in *A. grandis* seems to be similar to other insects in the steps of the process like dsRNA cleavage, dsRNA binding and Argonaute activity, but differs of dipterans in dsRNA uptake. No gene involved in dsRNA degradation was found.
(XLSX)
