# Supplementary figures and images for: Transcriptome Analysis in Cotton Boll Weevil (Anthonomus grandis) and RNA Interference in Insect Pests
Source: PLoS One. 2013 Dec 27;8(12):e85079. doi: 10.1371/journal.pone.0085079 (PMC3874031; doi:10.1371/journal.pone.0085079)

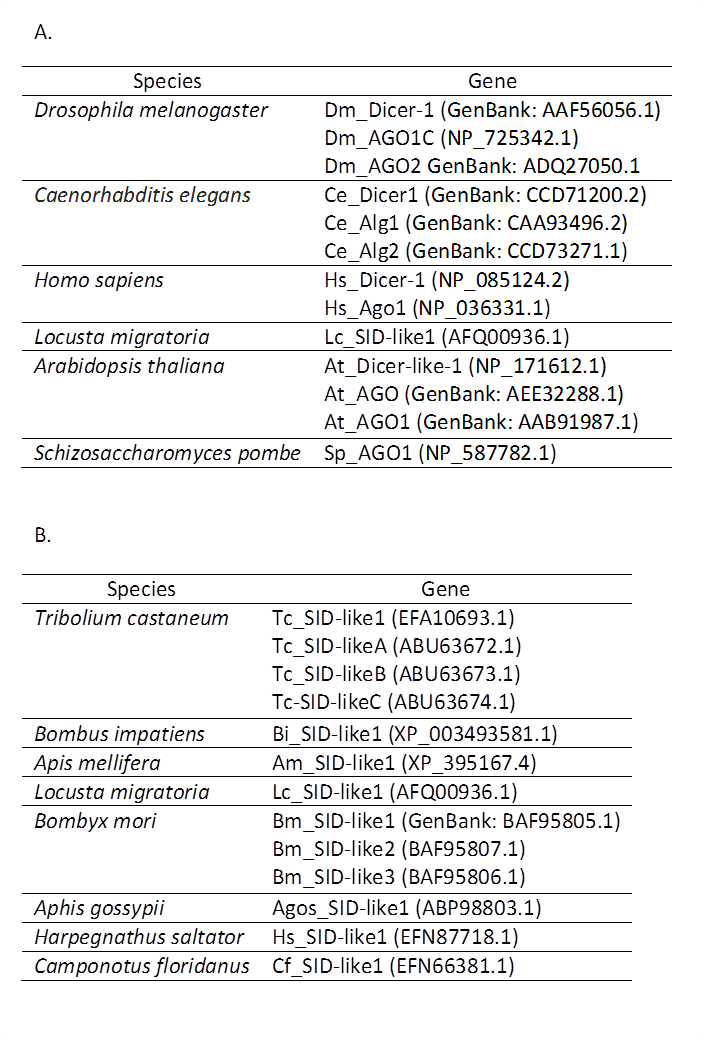

Supplement: Figure S1 — Orthologous genes used in PAZ Domain alignment (A) and SID-1 phylogenetic Analysis (B). Two largest cotton boll weevil PAZ Domain-containing contigs were selected for alignment with PAZ domains of argonautes and dicer-like proteins of other organisms including insects. For SID-like protein phylogenetic analysis, a cotton boll weevil complete gene sequence was translated and aligned to complete protein sequences. (TIF) [file pone.0085079.s001.tif]

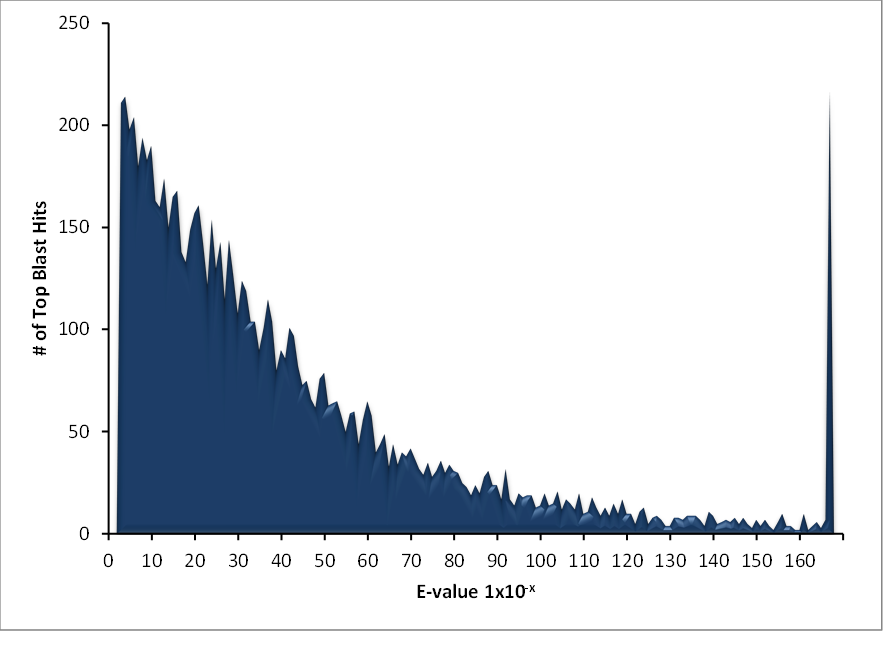

Supplement: Figure S2 — E-value for the top BLASTx hits. Sequences with e-value equal to 0 are represented in a peak at right. 84.9% of the contigs showed significant blast matches at a cutoff e-value ≤ 10-3. (TIF) [file pone.0085079.s002.tif]

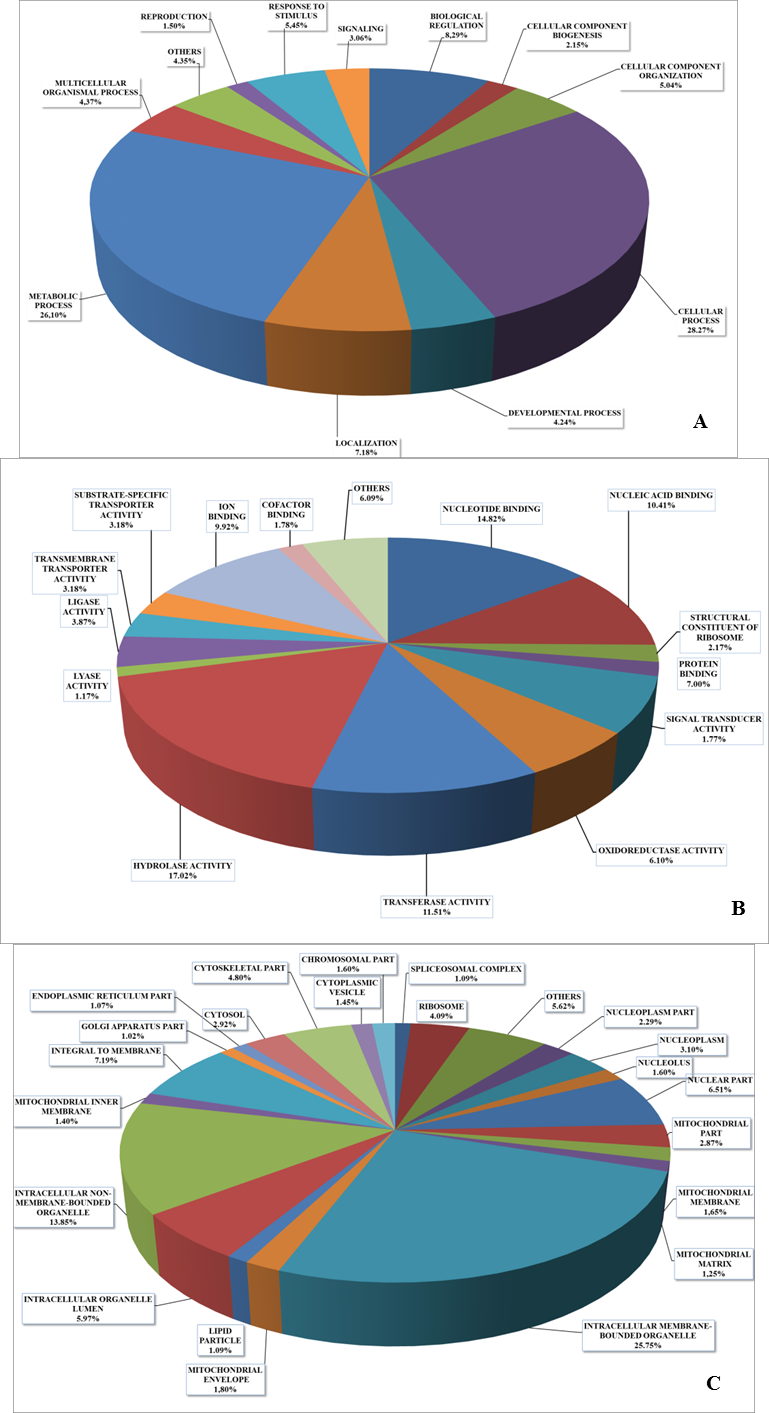

Supplement: Figure S3 — Gene ontology (GO) categories for A. grandis transcriptome. The terms were classified on level 2, 3 and 5 in the (A) Biological Process, (B) Cellular Component and (C) Molecular Function, respectively. The dominant terms for Molecular function are transporter activity and binding, while the dominant term for Biological process is pigmentation. Within Cellular component the dominant terms are evenly divided between organelle, cell part and organelle part. The percentage of contigs in each GO term is shown. (TIF) [file pone.0085079.s003.tif]
